# Knowledge, Attitudes and Practices Regarding Venous Thromboembolism Prevention Among Orthopaedic Nurses in Comprehensive Hospitals: A Cross‐Sectional Survey

**DOI:** 10.1111/ijn.70048

**Published:** 2025-08-28

**Authors:** Wenwen Cui, Neng Ru, Huali Guo, Li Song, Ying Cui, Jingjing Li, Feifan Wang

**Affiliations:** ^1^ The First College of Clinical Medical Science China Three Gorges University Yichang China; ^2^ Yichang Central People's Hospital Yichang China

**Keywords:** attitudes, knowledge, nurses, orthopaedic, practices, venous thromboembolism

## Abstract

**Aims:**

This study aimed to investigate the current status of knowledge, attitudes and practices regarding venous thromboembolism (VTE) prevention among orthopaedic nurses in comprehensive hospitals in Yichang, Hubei Province, China, and to analyse influencing factors to provide a reference for implementing VTE prevention strategies.

**Methods:**

From February to March 2024, a total of 257 orthopaedic nurses from nine comprehensive hospitals in Yichang were surveyed using a convenience sampling method and investigated with a knowledge, attitudes and practices questionnaire for VTE prevention. Multiple linear regression analysis was conducted to identify factors influencing knowledge, attitudes and practices regarding VTE prevention among orthopaedic nurses.

**Results:**

The overall score for knowledge, attitudes and practices regarding VTE prevention among orthopaedic nurses was 88.54 ± 5.73, with knowledge scoring 73.56 ± 7.69, attitudes scoring 95.66 ± 7.79 and practices scoring 95.96 ± 7.04. Position, training frequency, quality inspection and discharge follow‐up were significant factors influencing knowledge, attitudes and practices scores (*p* < 0.05).

**Conclusions:**

In the orthopaedic departments of comprehensive hospitals in Yichang, Hubei Province, China, the overall level of knowledge, attitudes and practices regarding VTE prevention among nurses is generally good, with positive attitudes and practices observed. However, there is potential for improvement in knowledge levels. Recommendations include enhancing standardized VTE training, improving knowledge levels, strengthening health education and ensuring discharge follow‐up to optimize VTE prevention efforts.

## Summary

**What is already known about this topic?:**

- VTE is one of the most serious complications in orthopaedics, significantly affecting patient prognosis and imposing substantial costs on the healthcare system.
- Nurses play a critical role in VTE prevention, and their knowledge, attitudes and practices reflect their clinical nursing abilities.

**What this paper adds?:**

- Investigating the current state of knowledge, attitudes and practices regarding VTE prevention among orthopaedic nurses in nine comprehensive hospitals in Yichang City, findings indicate positive attitudes and practices but a lack of knowledge.
- Key influencing factors include position, training frequency, discharge follow‐up and quality inspection.

**The implications of this paper:**

- This study identifies factors influencing knowledge, attitudes and practices levels among orthopaedic nurses in Yichang comprehensive hospitals.
- The results of this study can provide reference data for clinical VTE prevention initiatives.

## 1 Introduction

Venous thromboembolism (VTE) refers to abnormal coagulation within veins, leading to partial or complete vascular obstruction and resulting in impaired venous return. It encompasses two types: deep vein thrombosis (DVT) and pulmonary thromboembolism (PTE) (Chinese Orthopaedic 2016). VTE represents a common complication during the perioperative period of major orthopaedic surgeries, contributing significantly to perioperative mortality and unexpected in‐hospital deaths among patients (Orthopedic Expert Group of the National Health Commission Enhanced Recovery After Surgery Expert Committee and Orthopedic Enhanced Recovery After Surgery Professional Committee of the Chinese Research Hospital Association 2022). Postoperative VTE in orthopaedic patients significantly impacts their quality of life, prolongs hospital stays, increases hospitalization costs and increases mortality rates (Shahi et al. 2017).

Guidelines indicate that VTE poses a significant risk to healthcare quality and patient safety. Improving the standardized prevention rate of VTE is also one of the goals for enhancing national medical quality and safety (Lei and Zhuang 2023). Following VTE prevention measures, the rates of VTE prophylaxis vary widely between institutions and nations, falling between 13% and 70% on average (Ghulam et al. 2024).

In the complex field of healthcare, nurses, as the primary caregivers, play an indispensable and crucial role in the process of the prevention and treatment of VTE. Their professional knowledge levels enable them to closely monitor patients' conditions, offer timely preventive measures and collaborate effectively with other medical staff to safeguard patients from the potential threats of VTE (Wang et al. 2017; Abuzied et al. 2022; Abuzied et al. 2024; Tolera and Gebremedhin 2024).

This study therefore aimed to investigate the knowledge, attitudes and practices (KAP) regarding VTE prevention among orthopaedic nurses in nine comprehensive hospitals in the Yichang area. It aimed to analyse influencing factors and propose strategies with the aim of providing a basis for the implementation of VTE prevention measures.

## 2 Materials and Methods

### 2.1 Participants

A cross‐sectional survey study was conducted between February and March 2024. Using convenience sampling, orthopaedic nurses were recruited from nine comprehensive hospitals in Yichang, Hubei Province. Inclusion criteria were as follows: (1) possession of a nursing licence and engagement in frontline clinical work in orthopaedics; (2) minimum nursing experience of 1 year; and (3) voluntary participation with informed consent. Exclusion criteria were (1) nursing interns, nurse practitioners or nursing assistants and (2) nurses on study leave or vacation during the survey period.

### 2.2 Research Tools

A questionnaire on knowledge, attitudes and practices regarding VTE prevention was developed through literature review, consultation of clinical guidelines and discussions with orthopaedic experts within the hospital (Huang et al. 2018; Lei and Zhuang 2023; Tolera and Gebremedhin 2024). The questionnaire consisted of two parts.

The first part included general information such as gender, education level, professional title, work experience, hospital level, frequency of participation in VTE‐related training, presence of VTE‐related quality inspection and whether follow‐up is conducted for discharged high‐risk or diagnosed VTE patients.

The second part consisted of a questionnaire on VTE prevention, encompassing three dimensions: knowledge, attitudes and practices: (1) The knowledge dimension comprised 35 items, answered in a true‐or‐false format. Each correct answer earned one point, whereas incorrect or unknown responses scored zero. Scores ranged from 0 to 35, with higher scores indicating a better understanding of VTE‐related knowledge. (2) The attitudes dimension consisted of eight items, all positively scored using a Likert 5‐point scale ranging from *strongly disagree* to *strongly agree*, scored from 1 to 5, respectively. Scores ranged from 8 to 40, with higher scores indicating a more proactive attitude among surveyed nurses towards preventing VTE occurrence in patients. (3) The practices dimension comprised a total of 13 items, rated 1–5 on a scale of *never*, *occasionally*, *sometimes*, *frequently* and *always*. Scores ranged from 13 to 65, with higher scores indicating better execution of nursing measures for VTE prevention. The total score ranged between 21 and 140 points. Scores for each item, scores for each dimension and the total score were converted to a percentage basis: Standard score = (average score/total score) × 100%. Scores above 85 were considered good, scores between 60 and 85 were deemed passable and scores below 60 were regarded as inadequate (Xie et al. 2022). The Cronbach's alpha coefficient of the questionnaire was 0.925, the Kaiser–Meyer–Olkin (KMO) measure of sampling adequacy was 0.895 (*p* < 0.001), and the cumulative variance explained 70.032%. These results indicate good reliability and validity.

### 2.3 Data Collection Methods

During the period of February to March 2024, a questionnaire was delivered to orthopaedic nurses from nine comprehensive hospitals in the Yichang region of Hubei Province, leveraging the Yichang Orthopedic Medical Quality Control Center. The survey elaborated on the purpose and significance of the study, aiming to secure comprehension and support. The questionnaire was distributed via QR codes using ‘Wenjuanxing’, a professional online questionnaire platform. Through this platform, users easily create, publish and manage various types of questionnaires, accessing functions equivalent to Amazon Mechanical Turk. Respondents scanned the code online and filled out the questionnaire according to the provided instructions. The online questionnaire platform was closed after 2 months. The survey was conducted anonymously, and all questions required completion to enable submission. With 71 questions for nurses to complete in the questionnaire, in order to avoid including questionnaires where responses had been entered without thinking, which would affect the quality of the questionnaire, surveys with uniform responses or completed in ≤ 2 min were deemed invalid and excluded. Additionally, each IP address was restricted to one response to prevent duplicate submissions, ensuring the accuracy and validity of the survey results. A total of 257 questionnaires were collected for this survey, all of which were deemed valid. With 265 eligible nurses in the hospital, this represented a 96.98% response rate.

### 2.4 Statistical Methods

The collected data underwent dual verification and was then entered into Excel. Subsequently, SPSS 25.0 was utilized for data processing and analysis. Descriptive statistics were employed to analyse the general characteristics of the participants. The scores for the knowledge, attitudes and practices of the surveyed nurses were presented as mean ± standard deviation. Single‐factor analysis of variance (ANOVA) or *t* tests were employed to analyse the impact of various factors on the knowledge, attitudes and practices scores related to VTE prevention. Variables showing significant associations (*p* < 0.05) in the univariate analysis were included in the multivariate analysis. To identify independent factors influencing orthopaedic nurses' knowledge, attitudes and practices regarding VTE prevention, a multiple linear regression analysis was performed. A significance level of *p* < 0.05 was utilized to determine statistically significant differences.

### 2.5 Ethical Consideration

This study was conducted in accordance with the Declaration of Helsinki and approved by the First College of Clinical Medical Science, China Three Gorges University Ethics Committee (2024‐049‐02) for studies involving humans. Informed consent was obtained from all subjects involved in the study.

## 3 Results

1 The scores for knowledge, attitudes and practices regarding VTE prevention were presented in Table 1

**TABLE 1:** Survey on knowledge, attitudes and practices scores of VTE prevention (*n* = 257).

VTE prevention KAP	$\bar{x}\pm s$, score	Good	Qualified	Unqualified
Knowledge	73.56 ± 7.69	27 (10.50%)	225 (87.55%)	5 (1.95%)
Attitudes	95.66 ± 7.79	225 (87.55%)	31 (12.06%)	1 (0.39%)
Practices	95.96 ± 7.04	222 (86.38%)	35 (13.62%)	0 (0%)
Total score	88.54 ± 5.73	202 (78.60%)	54 (21.01%)	1 (0.39%)

This survey's findings indicate that nurses' attitudes, practices and total score regarding VTE prevention are generally at a good level. However, the knowledge dimension score 74.56 ± 7.69 is only at a qualified level. Among the 257 orthopaedic nurses surveyed, only 10.5% scored well in knowledge, whereas 1.95% scored poorly. Findings indicated a need to strengthen nurses' knowledge regarding VTE prevention.

2 The five items with the lowest scores in each dimension of the scale of knowledge, attitudes and practices regarding VTE prevention are shown in Table 2

**TABLE 2:** The five lowest scoring items across dimensions in the VTE prevention KAP scale.

Dimension	Entry items	Score
Knowledge	VTE includes deep vein thrombosis (DVT), muscular vein thrombosis (MCVT) and pulmonary thromboembolism (PTE).	0.05 ± 0.22
	The primary causes leading to VTE are venous stasis or sluggish blood flow, venous endothelial injury and hyperlipidaemia.	0.08 ± 0.28
	It is recommended to wear gradient pressure stockings during the daytime and refrain from wearing them at night. Regular assessment of patient limbs and usage is advised.	0.06 ± 0.25
	The postoperative period within 8 h is the high‐risk period for VTE formation during orthopaedic surgery.	0.12 ± 0.33
	For patients with acute lower limb DVT, routine use of inferior vena cava filters is recommended.	0.04 ± 0.20
Attitudes	You believe that VTE is preventable.	4.79 ± 0.41
	You think it is necessary to formulate and implement a VTE prevention and treatment workflow.	4.84 ± 0.39
	You consider it necessary to include VTE prevention in quality checks.	4.76 ± 0.55
	You believe that you have sufficient expertise to instruct patients to prevent VTE formation.	4.59 ± 0.67
	You think it is necessary to train medical staff about VTE prevention.	4.82 ± 0.39
Practices	After admission, I proactively evaluate and identify high‐risk patients.	4.71 ± 0.67
	I can correctly use the VTE risk assessment form to risk screen patients.	4.65 ± 0.67
	I can perform VTE risk assessments as frequently as recommended in guidelines (within 24 h after admission, when the condition or treatment changes, and within 24 h before discharge).	4.68 ± 0.71
	I will improve the bleeding risk assessment for patients at moderate and high risk of VTE.	4.58 ± 0.88
	When patients are at high risk for VTE, I can communicate with doctors/nurses in a timely manner.	4.72 ± 0.65

### 3.1 Analysis of Factors Influencing KAP in VTE Prevention

#### 3.1.1 Univariate Analysis

This study examined the demographic and professional characteristics of 257 orthopaedic nurses, including their highest education level, professional title, years of work experience, hospital level, position and frequency of training, quality inspections and discharge follow‐up. Detailed results are presented in Table 3. There was a statistically significant difference in the total score of knowledge, attitudes and practices regarding VTE prevention based on the frequency of participation in VTE‐related training and whether follow‐up visits were conducted for high‐risk or diagnosed VTE patients upon discharge (*p <* 0.05). Additionally, educational level, professional title, years of work experience, position and frequency of participation in VTE‐related training showed statistically significant differences in VTE prevention knowledge (*p* < 0.05). Moreover, years of work experience, frequency of participation in VTE‐related training, follow‐up visits for high‐risk or diagnosed VTE patients upon discharge and whether the hospital had VTE‐related quality inspections exhibited statistically significant differences in VTE prevention attitudes (*p* < 0.05). Lastly, follow‐up visits for high‐risk or diagnosed VTE patients upon discharge showed statistically significant differences in VTE prevention practices (*p* < 0.05).

**TABLE 3:** Univariate analysis of nurses' VTE prevention KAP based on demographic characteristics (*n* = 257).

Item	*n*	Knowledge score $\bar{x}\pm s$	*t*/*F*	*p*	Attitudes score $\bar{x}\pm s$	*t*/*F*	*p*	Practices score $\bar{x}\pm s$	*t*/*F*	*p*	Total score $\bar{x}\pm s$	*t*/*F*	*p*
Highest level of education			4.068	0.018*		0.647	0.590		1.139	0.322		0.727	0.485
Associate degree	55	71.64 ± 8.23			94.32 ± 10.81			97.00 ± 6.30			87.77 ± 6.65		
Bachelor's degree	200	73.99 ± 7.43			96.08 ± 6.67			95.64 ± 7.25			88.74 ± 5.04		
Master's degree	2	84.29 ± 6.06			90.77 ± 13.05			100 ± 0.00			89.64 ± 8.59		
Professional title			4.164	0.017*		0.009	0.991		1.375	0.271		0.786	0.457
Junior	120	72.21 ± 6.38			95.59 ± 8.99			95.50 ± 7.71			88.13 ± 5.67		
Intermediate	128	74.55 ± 8.56			95.72 ± 6.62			96.25 ± 6.54			88.83 ± 5.31		
Senior	9	77.46 ± 7.88			95.73 ± 6.38			98.06 ± 4.10			95.96 ± 7.04		
Work experience			2.763	0.028*		3.646	0.009**		1.467	0.222		1.178	0.329
≤ 5 years	39	71.58 ± 6.68			97.40 ± 5.94			95.19 ± 8.72			88.81 ± 4.13		
6–10 years	74	72.74 ± 6.30			94.37 ± 10.77			95.10 ± 7.47			87.56 ± 6.69		
11–15 years	93	75.58 ± 7.82			94.79 ± 6.86			96.16 ± 6.79			88.59 ± 5.33		
16–20 years	12	73.33 ± 9.92			97.05 ± 3.79			97.92 ± 3.82			89.23 ± 4.19		
> 20 years	39	72.38 ± 9.18			98.03 ± 4.19			97.31 ± 5.48			89.78 ± 4.28		
Hospital level			0.560	0.576		−1.547	0.123		−0.618	0.537		−1.105	0.270
Tertiary hospital	107	73.24 ± 6.66			96.51 ± 6.42			96.29 ± 6.96			88.98 ± 5.12		
Secondary hospital	150	73.79 ± 8.36			95.06 ± 8.60			95.73 ± 7.12			88.22 ± 5.65		
Position			−3.794	< 0.001***		0.979	0.329		−0.639	0.524		−0.929	0.354
Yes	21	81.09 ± 9.67			94.07 ± 8.26			96.91 ± 5.96			89.59 ± 5.75		
No	236	72.89 ± 7.14			95.80 ± 7.75			95.88 ± 7.14			88.44 ± 5.41		
Training frequency			3.409	0.035*		3.700	0.026*		2.047	0.135		6.367	0.003**
0	6	61.43 ± 15.62			94.87 ± 8.46			96.67 ± 8.17			85.12 ± 7.65		
1–2	76	72.52 ± 5.92			93.34 ± 8.78			94.84 ± 7.97			86.78 ± 5.80		
3–4	55	73.09 ± 7.77			95.30 ± 6.73			94.96 ± 7.41			87.97 ± 4.72		
≥ 5	120	75.05 ± 7.55			97.33 ± 7.21			97.10 ± 6.03			90.08 ± 4.97		
Quality inspection			−0.058	0.955		2.690	0.031*		0.010	0.992		2.345	0.051
Yes	249	73.55 ± 7.18			96.16 ± 6.84			95.96 ± 7.04			88.80 ± 5.02		
No	8	73.93 ± 18.29			80.19 ± 16.74			95.94 ± 7.78			80.27 ± 10.25		
Discharge follow‐up			−1.413	0.159		4.089	< 0.001***		3.189	0.003**		4.616	< 0.001***
Yes	220	73.29 ± 7.48			96.75 ± 6.56			96.65 ± 6.51			89.32 ± 4.71		
No	37	75.21 ± 8.78			89.19 ± 10.92			91.89 ± 8.67			83.84 ± 6.96		

* *p* < 0.05.

** p < 0.01.

*** *p* < 0.001.

#### 3.1.2 Multivariate Analysis

To control for confounding factors, we set the scores of nurses' knowledge, attitudes and the total score of KAP as dependent variables separately. Then, we conducted multiple linear stepwise regression analyses with significant factors from the univariate analyses set as independent variables.

Position and training frequency were identified as the primary factors influencing knowledge scores (*p* < 0.05). For attitudes dimension scores, the three main factors are whether postdischarge follow‐up was conducted for VTE high‐risk and diagnosed patients, the frequency of participation in VTE‐related training and the presence of VTE‐related quality inspections (*p* < 0.05). Regarding the total score of knowledge, attitudes and practices regarding VTE prevention, the two main factors are the frequency of participation in VTE‐related training and whether postdischarge follow‐up was conducted for VTE high‐risk and diagnosed patients (*p* < 0.05). Please refer to Table 4.

**TABLE 4:** Multiple linear regression analysis of nurses' KAP regarding VTE prevention (*n* = 257).

Dependent variable	Independent variable	Regression coefficient	Standard error	Standardized regression coefficient	*t*	*p*
Nurse VTE prevention knowledge^a^	Constant	62.485	3.913	—	15.968	< 0.001***
	Position	6.714	1.763	0.240	3.808	< 0.001***
	Training frequency	1.773	0.504	0.212	3.518	0.001**
Nurse VTE prevention attitudes^b^	Constant	109.375	3.317	—	32.974	< 0.001***
	Discharge follow‐up	−5.664	1.307	−0.256	−4.334	< 0.001***
	Training frequency	1.117	0.491	0.132	2.273	0.024*
	Quality inspection	−11.828	2.655	−0.264	−4.456	< 0.001***
Total score for nurse VTE prevention^c^	Constant	89.761	1.548	—	57.991	< 0.001***
	Training frequency	1.485	0.336	0.251	4.421	< 0.001***
	Discharge follow‐up	−5.126	0.877	−0.332	−5.845	< 0.001***

*Note:* (1) ‘Position’ is a binary variable. The reference category is ‘not having a position’, whereas the comparison category is ‘having a position’. (2) ‘Training frequency’ is a multicategory variable, with categories included 0, 1–2, 3–4 and ≥ 5 times. No specific single reference category was defined; instead, each category was compared independently. (3) ‘Discharge follow‐up’ is a binary variable. The reference category is ‘follow‐up action taken’, whereas the comparison category is ‘no follow‐up action taken’. (4) ‘Quality inspection’ is a binary variable. The reference category is ‘presence of VTE‐related quality checks’, whereas the comparison category is ‘absence of VTE‐related quality checks’.

^a^ *R* = 0.376, adjusted *R*‐square = 0.124, *F* = 8.279, *p <* 0.001.

^b^ *R* = 0.464, adjusted *R*‐square = 0.203, *F* = 17.253, *p* < 0.001.

^c^ *R* = 0.434, adjusted *R*‐square = 0.182, *F* = 29.476, *p <* 0.001.

* *p* < 0.05.

** *p* < 0.01.

*** *p* < 0.001.

In the multivariate analysis results, there were strong and complex associations between each variable and knowledge, attitude and practice. For the variable of ‘position’, the analysis showed that it had a significant positive correlation with the VTE prevention and treatment knowledge score of nurses. For the variable of ‘training frequency’, the analysis showed that it was significantly and positively correlated with the scores of knowledge, attitude and practice of VTE prevention. With the increase of training frequency, the knowledge score of nurses was significantly improved, indicating that more frequent training can effectively improve nurses' mastery of VTE prevention and treatment knowledge. In terms of attitudes, the increase of training frequency also promoted the positive attitudes of nurses towards VTE prevention and treatment, and the score of positive attitudes increased accordingly. In the practices dimension, the frequency of training was also positively correlated with the practice score, which means that nurses who have undergone multiple training can perform VTE prevention and control measures more standardized and effectively in practical work. For the variable of ‘discharge follow‐up’, the analysis showed that it was associated with increased scores of VTE prevention attitudes and practices. For the variable of ‘quality control inspection’ and VTE prevention attitudes, VTE‐related quality inspection through the supervision and feedback of nurses' work can promote nurses to pay more attention to the standardization and accuracy of VTE prevention work and can also strengthen nurses' attention to VTE prevention work and promote them to treat prevention work with a more positive attitude. Therefore, VTE related quality examination is associated with an increase in the score of VTE prevention attitudes.

## 4 Discussion

### 4.1 The Overall Level of KAP Regarding VTE Prevention Among Orthopaedic Nurses at Comprehensive Hospitals in Yichang Is Satisfactory, Although There Is a Deficiency in Knowledge

In this study, the items with the lowest scores in the attitudes and practices dimensions of VTE prevention still received high scores, indicating that orthopaedic nurses performed well in these aspects. The five items with the lowest scores in the knowledge dimension were the VTE definition, aetiology, physical prevention measures, high‐risk factors and acute management, suggesting a lack of relevant knowledge. This finding is consistent with the findings of Wang et al. (2019, 2021). Knowledge of VTE prevention serves as the foundation for implementing VTE‐related clinical practices, which also indicates that nurses need to do better to make up for the shortcomings in knowledge learning. In VTE prevention, doctors and nurses have not yet established a collaborative and complementary working mode. Their focus is limited to knowledge related to medical and nursing procedures, which is attributed to a lack of systematic training (Zhai et al. 2019; Wei et al. 2021). Studies have shown that the consistency of VTE risk assessment among medical staff is poor, and it is necessary to systematically train medical staff. It is important to establish a standardized evaluation process and build a scientific and effective VTE prevention and treatment system (Geng et al. 2023). This finding is consistent with the findings of Zhou et al. (2023). The enthusiasm of orthopaedic nurses to participate in VTE prevention, in terms of both attitudes and practices, should be transformed into the motivation for learning knowledge, so that the three aspects of knowledge, attitudes and practices regarding VTE prevention can truly form a virtuous circle.

### 4.2 Analysis of Factors Influencing KAP of VTE Prevention Among Orthopaedic Nurses at Yichang Comprehensive Hospitals

As shown in Table 4, position influence is an independent factor affecting the knowledge dimension (*p* < 0.05); quality inspections are independent factors affecting the attitudes dimension (*p* < 0.05); training frequency is an independent factor affecting the knowledge, attitudes and total score dimensions (*p* < 0.05); and discharge follow‐up is an independent factor affecting the attitudes and total score dimensions (*p* < 0.05).

#### 4.2.1 Position as an Independent Influencing Factor in the Dimension of Knowledge

Position is an independent influencing factor in the dimension of knowledge. Among the 257 orthopaedic nurses in this study, 21 of them held positions and were the head nurses of their respective departments. The results indicate that nurses with position (head nurses, 8.2%) score significantly higher than those without position (91.8%), demonstrating better mastery of VTE prevention knowledge. As they assume leadership responsibilities and a leading clinical role, they need to master VTE prevention knowledge more deeply and pay more attention to the management and supervision of VTE prevention in their daily work, so they scored relatively high in VTE prevention knowledge. VTE prevention and treatment are comprehensive goals of hospital medical quality management and control, as well as important criteria for hospital grading reviews. As nursing managers, they attach great importance to VTE prevention work, possess rich knowledge accumulation and clinical experience and have more opportunities to participate in national or regional training courses and academic seminars. This broadens their channels for acquiring knowledge, enabling them to gain more VTE‐related knowledge (Feng et al. 2022).

#### 4.2.2 Quality Inspection Stands as an Independent Influencing Factor in the Dimension of Attitude

Nursing quality inspection is an important part of clinical nursing work. Nursing managers in different hospitals will set up evaluation tables for the three‐level nursing quality control management mode of VTE prevention based on their own clinical situations and formulate nursing quality management standards and inspection methods. In this study, orthopaedic nurses believe that it is necessary to incorporate VTE prevention into quality inspection. However, currently, there is no homogeneous management model across multiple hospitals, and there is a lack of a standardized VTE inspection process for daily supervision. Therefore, there are some differences in the content and links of VTE prevention quality control among nine comprehensive hospitals, and nurses' knowledge, attitudes and practices regarding VTE prevention also vary. The implementation of homogeneous management in different nursing units of multiple hospitals can effectively improve the quality of nursing and patient satisfaction and enhance nurses' awareness of active service (Hou et al. 2020). It is also worthy of being learned from in the quality inspection of VTE prevention, so as to increase nurses' attention to VTE prevention and promote the improvement of clinical VTE nursing quality (Ouyang and Feng 2021).

#### 4.2.3 The Frequency of Training Sessions Constitutes an Independent Determinant Influencing the Dimensions of Knowledge, Attitudes, and KAP

The frequency of orthopaedic nurses' participation in VTE‐related training sessions is positively correlated with their knowledge, attitudes and overall competency in VTE prevention, likely because in‐depth training enhances their awareness of VTE's clinical significance, thereby increasing professional motivation. As a key professional development mechanism, structured training provides nurses with both evidence‐based theoretical knowledge and practical clinical skills, promoting standardized care practices (Wu et al. 2018; Dai et al. 2020), with the ultimate goal of improving patient outcomes. Targeted education enables orthopaedic nurses to establish a virtuous cycle integrating knowledge, attitudes and practices (KAP) in VTE prevention, thereby strengthening evidence‐based clinical performance (Hao 2020). However, significant heterogeneity persists in current training approaches across institutions, ranging from fragmented online modules to purely observational or lecture‐based formats, highlighting the need for more systematic, guideline‐based training frameworks that enhance interdisciplinary collaboration between medical and nursing teams.

#### 4.2.4 Discharge Follow‐Up Serves as an Independent Influencing Factor in Both the Attitudes Dimension and the KAP Continuum

Discharge follow‐up is an integral part of continued care. Through follow‐up, healthcare providers can assess patients' postdischarge conditions, as well as their compliance and satisfaction with various treatment and care measures (Abuzied et al. 2021). Whether high‐risk patients receive discharge follow‐up also reflects the level of importance healthcare professionals attach to VTE. By discharge follow‐up of high‐risk or confirmed VTE patients, nurses can more intuitively see the impact of VTE prevention measures on the prognosis of patients, which will enhance their attention to VTE prevention and improve prevention attitudes. At the same time, in order to ensure the follow‐up effect, nurses will implement VTE prevention measures more standardized in their daily work, which helps to improve their preventive practices score. Currently, telephone follow‐up is the primary method used for discharge follow‐up (Alshammary et al. 2021). For patients at high risk of VTE, follow‐up and health education are conducted at 15, 30 and 90 days after discharge. Although nurse‐led follow‐up can enhance patients' attention to VTE prevention and provide guidance on medication and follow‐up appointments (Woods et al. 2019), the uneven levels of VTE awareness among healthcare providers in different medical institutions, coupled with the lack of standardized and systematic VTE follow‐up systems, result in incomplete information collection and inadequate assessment due to biases in understanding and cognition (Liu et al. 2021). This makes it challenging to detect VTE risks in patients early.

#### 4.2.5 The Key Role of Orthopaedic Nurses in VTE Prevention and Management

In 2015, the International Society on Thrombosis and Haemostasis, along with the World Thrombosis Experts Committee, included VTE as a special cause of mortality in the World Health Organization's (WHO) global study on the burden of diseases. With the introduction of China's first Guidelines for the Prevention and Treatment of Thrombotic Diseases, the updated Recommendations for the Prevention and Management of VTE in Hospitals and the nationwide establishment of model thrombosis prevention and treatment centres, both the government and healthcare institutions have prioritized comprehensive VTE prevention and management. Its development presents both challenges and opportunities (Committee for the Prevention and Treatment of Thromboembolic Diseases, Chinese Medical Association 2018). Due to the characteristics of orthopaedic diseases, surgical trauma and early postoperative limb immobilization, orthopaedics is a high‐incidence department for VTE. Nurses, as the primary caregivers for patients, play a crucial role in VTE prevention. Only by fully understanding the current state of knowledge, attitudes and practices regarding VTE among orthopaedic nurses can target improvements be made in the mastery of VTE knowledge and the implementation of prevention and treatment measures (Shaaban et al. 2021). In 2021, the release of the ‘Guidelines for the Prevention of Venous Thromboembolism in Perioperative Patients with Traumatic Orthopedics in China (2021)’ advocated for the establishment of a scientific and standardized medical care management system for thrombosis prevention, aiming to enhance the effectiveness of VTE prevention and treatment in orthopaedics and reduce the incidence and mortality of VTE among hospitalized patients (Lin et al. 2021). Orthopaedic nurses participate in the entire process management by conducting VTE‐related risk assessments, standardized screenings, diligent monitoring, early warning reports and active interventions for admitted patients, through to follow‐up and supervision upon discharge. The collaboration between medical and nursing staff promotes an integrated medical‐nursing thrombosis prevention system, adhering to the principle of early detection and early treatment, which can reduce the incidence and related mortality of VTE (Hongfei et al. 2019; Liling et al. 2024). The efforts of nurses in preventing VTE have been recognized, and only by continuously improving their own knowledge, attitudes and practice levels can they play a significant role in clinical practice.

### 4.3 Limitations

This study is limited by its cross‐sectional design and regional focus. Future research should expand both the geographical scope and sample size to comprehensively understand VTE prevention among nurses in Yichang hospitals, which would lay a foundation for standardized VTE prevention and management there, thereby contributing to the continuous improvement of clinical nursing quality. Additionally, this study excluded nurses with less than 1 year of experience, nursing interns, nursing assistants and so on. This may result in an unrepresentative sample of VTE prevention involved nursing staff, overlooking their key roles. Clinically, these excluded nurses have VTE prevention duties, with practices and preventive measure implementations differing from those in the study. Significantly, they can detect subtle patient symptoms in a unique way, providing clues for early VTE detection. Through frequent patient–family interactions, they build trust and obtain relevant first‐hand data. Therefore, subsequent research should incorporate them to fully understand VTE prevention and analyse their roles by experience level to support clinical optimization.

## 5 Conclusions

In conclusion, the overall level of knowledge, attitudes and practices regarding VTE prevention among orthopaedic nurses in comprehensive hospitals in the Yichang region of Hubei Province is satisfactory. Nurses demonstrate positive attitudes and practices towards VTE prevention, but there is room for improvement in their knowledge level. Training frequency, quality inspections, position and discharge follow‐up for high‐risk patients are influencing factors in knowledge, attitudes and practices regarding VTE prevention among orthopaedic nurses. Hospitals should implement systematic, evidence‐based measures to enhance KAP levels among orthopaedic nurses, thereby enabling them to play a more significant role in VTE prevention.

## Author Contributions

*Conceptualization*: Feifan Wang and Wenwen Cui. *Methodology*: Neng Ru. *Software*: Neng Ru. *Validation*: Ying Cui and Jingjing Li. *Formal analysis*: Huali Guo. *Investigation*: Li Song. *Resources*: Ying Cui and Jingjing Li. *Data curation*: Huali Guo and Li Song. *Writing – original draft preparation*: Wenwen Cui. *Writing – review and editing*: Feifan Wang. *Visualization*: Neng Ru. *Supervision*: Feifan Wang. *Project administration*: Feifan Wang. *Funding acquisition*: Feifan Wang. All authors have read and agreed to the published version of the manuscript.

## Conflicts of Interest

The authors declare no conflicts of interest.

## Data Availability

The data that support the findings of this study are available from the corresponding author upon reasonable request.
